# A database of atmospheric inorganic nitrogen deposition fluxes in China from satellite monitoring

**DOI:** 10.1038/s41597-023-02607-z

**Published:** 2023-10-13

**Authors:** Qian Gao, Xiuying Zhang, Lei Liu, Xuehe Lu, Yingying Wang

**Affiliations:** 1 International Institute for Earth System Science, Nanjing University, Nanjing, 210023 China; 2 Jiangsu Center for Collaborative Innovation in Geographical Information Resource Development and Application, Nanjing, 210023 China; 3 College of Earth and Environmental Sciences, Lanzhou University, Lanzhou, 730000 China; 4 School of Geography Science and Geomatics Engineering, Suzhou University of Science and Technology, Suzhou, 215009 China; 5 Jiaxing City Land Space Planning Research Co., LTD, Jiaxing, 314006 China

**Keywords:** Environmental sciences, Atmospheric science

## Abstract

Over the past century, atmospheric inorganic nitrogen (IN) deposition to terrestrial ecosystems has significantly increased and caused various environmental issues. China has been one of the hotspot regions for IN deposition, yet limited data exist regarding IN deposition fluxes in China at the regional scale. In this study, based on NO_2_ and NH_3_ columns acquired by satellite sensors, coupled with atmospheric chemical transport model (CTM), mixed-effects model and site observations, we constructed regional-scale IN dry and wet deposition models respectively, and finally proposed a spatially explicit database of IN deposition fluxes in China. The database includes the dry, wet and total deposition fluxes in China during 2011–2020, and the data are presented in raster form with a resolution of 0.25° × 0.25°. Overall, the database is of great importance for monitoring and simulating the trends of IN deposition over a long time series in China.

## Background & Summary

Atmospheric nitrogen (N) deposition plays an important role in the biological N cycle, with anthropogenic emissions of inorganic nitrogen (IN) returning to the surface as atmospheric deposition. The sources of N compounds are intricate. Reduced N components (NH_3_, NH_4_^+^, etc.) mainly originate from agricultural fertilization and animal husbandry. In contrast, oxidized N compounds (NO, NO_2_, NO_3_^−^, etc.) primarily come from industry and fossil fuel combustion. Considerable spatial and temporal variations exist between the two sources. The increase in N deposition in the short term can enhance the ecosystem vitality, but a large amount will inevitably have an impact on the environment. Over the past two decades, extensive scientific research on acid deposition in China has played a pivotal role in shaping national policies aimed at combating acid rain pollution. These policies primarily centred on curbing emissions of sulfur dioxide (SO_2_) and nitrogen oxides (NOx), which are the main contributors to acid deposition. Thanks to the effectiveness of policy development, there has been a growing international focus on nitrogen deposition in recent years. Atmospheric chemistry transport model (CTM) simulations have revealed that the areas with high N depositions in China have increased significantly over the past 40 years^1^, which may have negative impacts on forest, grassland, agricultural, aquatic and coastal ecosystems^2^.

Inorganic nitrogen deposition is mainly evaluated by three methods: ground sites monitoring, CTM simulations, and quantitative remote sensing estimation. Compared to dry deposition, the observations on wet deposition at ground sites started earlier. The earliest observations on wet depositions in China can be traced back to the national acid rain monitoring network established by the National Environment Bureau of China from 1981 to 1983^3^. This network focused on observing acid rain, in which nitrogen wet deposition fractions (NO_3_^−^ and NH_4_^+^) were measured. After that, several other networks were established successively. Most of them were concentrated in urban areas^4^, which cannot reveal the regional characteristics well^5,6^. Subsequently, the National Nitrogen Deposition Monitoring Network (NNDMN) was established by China Agricultural University, which can obtain comprehensive observations of dry and wet nitrogen deposition across China, covering farmland, grassland, woodland and urban areas^7^. Based on ground-based monitoring sites, the average atmospheric IN deposition flux in China ranged from 20.4 to 40.0 kg N ha^−1^ yr^−1^ during the period of 2010 to 2020^7–9^.

The atmospheric CTMs, with the capability to establish a connection between nitrogen sources and sinks, are based on emission inventory and meteorological data to simulate IN deposition fluxes^10^. There have been several global-scale atmospheric chemistry models, such as GEOS-Chem and MOZART^11^. However, their coarse spatial resolution limits the ability to provide detailed information on the spatial variations of IN deposition. Furthermore, atmospheric CTMs have tended to underestimate wet deposition of nitrate-N in the Asian region^12^. The atmospheric IN deposition fluxes simulated by CTMs ranged from 7.9 to 18.1 kg N ha^−1^ yr^−1^ in China^10,13^. There existed a large gap in the estimates of IN deposition in China by CTMS and site observations. Consequently, it is urgent to develop a supplementary approach to unravel the spatiotemporal patterns of atmospheric IN at a regional scale^14^.

Satellite observations have the advantages of wide spatial coverage, strong periodic observation capability and high spatial resolution^15^. It can provide an effective method to estimate NO_2_ and NH_3_ columns^16^, and has been widely used to observe regional pollutant gases. Currently, satellite sensors capable of monitoring NO_2_ include GOME (Global Ozone Monitoring Experiment), SCIAMACHY (SCanning Imaging Absorption spectroMeter for Atmospheric CHartographY), OMI (Ozone Monitoring Instrument), GOME-2 (Global Ozone Monitoring Experiment-2) and TROPOMI (TROPOspheric Monitoring Instrument). In particular, the OMI sensor has a relatively high spatial resolution and long-term observations on NO_2_. Compared to NO_2_, the development of remote sensors on observing atmospheric NH_3_ has been more delayed. Presently, the atmospheric NH_3_ columns can be retrieved from TES (Tropospheric Emission Spectrometer), AIRS (Atmospheric Infrared Sounder), IASI (Infrared Atmospheric Sounding Interferometer) and CrIS (Cross-track Infrared Sounder)^17^.

The process of IN deposition includes dry and wet deposition. Atmospheric dry deposition refers to the process in which nitrogenous substances in the form of particulate matter and gases in the atmosphere are transported to the Earth’s surface by gravity, adsorption of particulate matter and direct reception by plant stomata in the absence of precipitation^18^. It is typically estimated using inferential model that take into account the near-surface concentration of nitrogenous substances and the deposition rate^19^. Wet deposition is the process by which soluble IN is adsorbed by water droplets aloft and then falls to the ground, mainly in the form of particulate matter, during precipitation events (rain, snow, fog, etc.).

In this study, yearly IN depositions across China were estimated based on the atmospheric nitrogenous substance concentrations from satellite observations. The models were initially constructed to estimate dry and wet IN deposition fluxes using remotely sensed NO_2_ and NH_3_ columns and an atmospheric CTM; then the estimation results on IN depositions were evaluated using the ground observations; and finally, the data of seven major components in the IN deposition fluxes during 2011- 2020 across China were provided.

## Methods

### Database structure

**Fig. 1:**
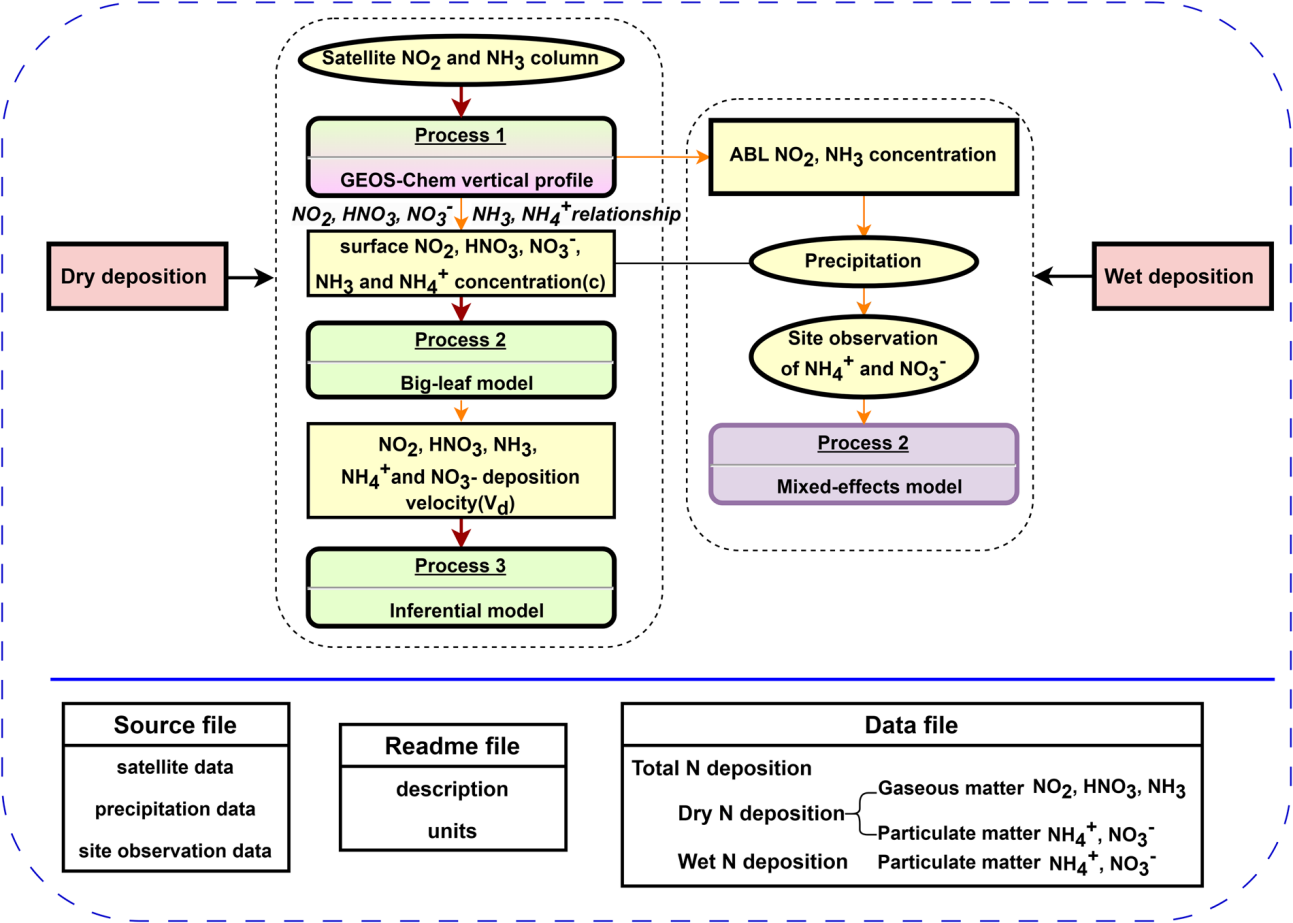
Flow chart of description of atmospheric inorganic nitrogen deposition and the composition of the database of IN deposition fluxes in China. Dry and wet deposition model construction consist of the following steps. Step 1: Process1 (GEOS-Chem vertical profile); Step 2 (Dry Deposition Model): Process2 (Big-leaf model) and Process3 (Inferential model); Step 2 (Wet Deposition Model): Process2 (Mixed-effects model). The database consists of three files: Readme file, Source file and Data file.

The atmospheric IN deposition fluxes database^20^ consists of three files (Fig. 1). The ‘data file’ provides yearly data for the dry, wet and total IN deposition fluxes. Specifically, dry deposition is the sum of two forms, gaseous and particulate, including NO_2_, HNO_3_, NH_3_, NO_3_^−^ and NH_4_^+^.Wet deposition is the sum of both NO_3_^−^-N and NH_4_^+^-N. The ‘readme file’ describes the ‘data file’ and the units of all variables included. The ‘source file’ includes the full references used in the database, which comprises satellite data and precipitation data.

### Data acquisition

The atmospheric nitrogen components are very complex, mainly including NH_3_, NO_2_, HNO_3_ gases, and particulate matter of NH_4_^+^ and NO_3_^−^. NO_2_ and NH_3_ are very critical precursors among the abundant IN components in the atmosphere. At present, the development of satellite technology has detected NO_2_ and NH_3_ concentrations in the atmosphere. These satellite observations could be used to estimate atmospheric IN deposition.

NO_2_ columns from OMI have been available since October, 2004. OMI is a UV-Visible wavelength spectrometer on the polar-orbiting NASA Aura satellite, launched on 15 July 2004, and follows a sun-synchronous orbit with an equator crossing time near 13:45, local time. In this study, the columnar NO_2_ is provided in the publicly released level 2.0 (http://www.temis.nl/), with an improved retrieval algorithm method (DOMINO 2.0) and the monthly tropospheric NO_2_ columns from Jan 2011 to Dec 2020 are used to estimate NO_2_ deposition fluxes, with a spatial resolution of 0.125° × 0.125°. Data quality control was used to select NO_2_ columns: (1) screen and exclude the image elements affected by OMI row anomalies; (2) select the image elements with less than 30% cloud inversion values to obtain as many valid image elements as possible.

We also used daily NH_3_ columns from the IASI during Jan. 2011 - Dec 2020 (http://cds-espri.ipsl.upmc.fr/etherTypo/index.php?id=1700&L=1). The data is in Network Common Data Form (NetCDF) format, with a unit of molec·cm^−2^ and a spatial resolution of 0.25° × 0.25°. The sensor provides global coverage twice a day, at 9:30 a.m. and 9:30 p.m. We used the daytime column, when there is a greater thermal contrast and greater sensitivity to atmospheric NH_3_^8^. In terms of data quality control, we required a cloudiness of less than 25% and a relative error lower than 100% or absolute error less than 5 × 15 molec·cm^−2^.

We interpolated to estimate the missing values for the collected NO_2_ and NH_3_ columns, respectively. For the NO_2_ columns, we resampled them to match the same spatial resolution as NH_3_. For the NH_3_ columns, we first synthesized the daily data to calculate the arithmetic average of monthly and yearly columns^17^. To generate continuous maps, we performed the Original Kriging interpolation to estimate the values at these cells with outlier values.

GEOS-Chem is a three-dimensional (3-D) global chemical transport model that simulates the chemical and transport processes. The source program is available free of charge from the GEOS-Chem Chemistry Model Group at Harvard University (http://acmg.seas.harvard.edu). For the emission inventory, the anthropogenic sources of NOx and NH_3_ were obtained from the monthly MIX inventory (http://meicmodel.org/).

The observation data of gaseous, particulate, wet N depositions at sites in China were collected as validation data from the NNDMN^7,21^. We used the ground-level NH_3_ and NO_2_ concentrations from 32 sites in 2014 to evaluate the performance of dry N deposition model; In wet deposition model, we used the measured wet NH_4_^+^ and NO_3_^−^ deposition from 32 sites during 2011–2012 to establish mixed-effects model^22^. In addition, the monthly precipitation data from 2011 to 2020 across China were provided by China Meteorological Administration (CMA). The detailed discussion on the data set and processing can be found at China Meteorological Data Sharing Service System (http://data.cma.cn/).

### Dry deposition model

The dry deposition model is constructed using an inferential method, expressed as^23^:

1 $${\rm{F}}={\rm{C}}\times {{\rm{V}}}_{{\rm{d}}}$$

Where C denotes satellite-derived surface NO_2_ and NH_3_ concentration and V_d_ indicates the rate of dry N deposition.

We used the stratified NO_2_ and NH_3_ concentrations simulated by GEOS-Chem to fit their contour functions in the vertical direction, which converts the column from satellite monitoring to those at surface level^24^. In this study, the 47 layers’ NO_2_ concentrations at 2° × 2.5° were used to construct the vertical profile models. Taking the approach to estimate surface NO_2_ concentrations as an example, steps are introduced in the following. The procedure to estimate the surface NH_3_ concentrations is similar to that.

1. Simulate NO_2_ concentrations in the atmosphere by GEOS-Chem. The hour NO_2_ concentrations at 47 vertical layer (from the Earth’s surface to the top of the stratosphere) at 00:00, 6:00, 12:00, 18:00 were simulated by GEOS-Chem and the daily NO_2_ concentrations at 12:00 were synthesized into monthly data.
2. Simulate the profile function to describe the vertical variations of NO_2_ concentrations. Since single Gaussian fitting may not capture the detail of vertical distribution of NO_2_ well^25,26^, we used multiple Gaussian functions to simulate the profile of the tropospheric NO_2_ concentrations^27^.
  2 $$\rho \left(Z\right)={\sum }_{i=1}^{n}{{\rho }_{max,i}e}^{-{\left(\frac{z-{z}_{0,i}}{\sigma }\right)}^{2}}$$
  Here, *ρ*(*Z*) represents the concentration of NO_2_ at a certain height level and *Z* is the height of a layer in the GEOS-Chem; n ranges from 2 to 6, representing the number of Gaussian items; *ρ*_*max,i*_, *z*_0*,i*_ and *σ* are the maximum NO_2_ concentration, the corresponding height with the maximum NO_2_ concentration and the thickness of the NO_2_ concentration layer. The determination coefficient of R^2^ and root-mean-square error (RMSE) were used to assess each model performance. The mode with the highest R^2^ and lowest RMSE (i.e., determined the value of n) was selected to describe the profile function.
  The NO_2_ columns in troposphere are simulated by integral calculation:
  3 $$\varphi ({h}_{{\rm{trop}}})={\int }_{0}^{{h}_{{\rm{trop}}}}\rho (Z){\rm{d}}x$$
  $\varphi \left({h}_{{\rm{trop}}}\right)$ denotes NO_2_ columns and *h*_trop_ indicates the tropospheric height.
3. Estimate the surface NO_2_ concentration based on the proportional of the near-surface to tropospheric concentrations obtained from both model simulations and satellite estimates.
  4 $${S}_{{\rm{G}}\_{{\rm{NO}}}_{2}}={S}_{{\rm{trop}}}\times \frac{\rho \left({h}_{{\rm{G}}}\right)}{\varphi \left({h}_{{\rm{trop}}}\right)}$$
  Where *S*_*G*_NO2_ and *S*_trop_ represent the surface NO_2_ concentration and the tropospheric column from satellite; and *ρ*(*h*_*G*_) and $\varphi \left({h}_{{\rm{trop}}}\right)$ represent the model-simulated NO_2_ concentration at surface level and tropospheric NO_2_ column, respectively.
4. Convert the instantaneous satellite-derived surface NO_2_ concentration to the daily average using the ratio of average surface NO_2_ concentration to that at satellite overpass time by GEOS-Chem.

5 $${S}_{{\rm{G}}\_{{\rm{NO}}}_{2}^{* }}=\frac{{G}_{{\rm{GEOS}} \mbox{-} {\rm{Chem}}}^{1-24}}{{G}_{{\rm{GEOS}} \mbox{-} {\rm{Chem}}}^{{\rm{overpass}}}}\times {S}_{{\rm{G}}\_{{\rm{NO}}}_{2}}$$

Where ${S}_{{\rm{G}} \mbox{-} {{\rm{NO}}}_{2}^{* }}$ and ${S}_{{\rm{G}} \mbox{-} {{\rm{NO}}}_{2}}$ are near-surface NO_2_ concentration of daily average and that at satellite overpass time. ${G}_{{\rm{GEOS}} \mbox{-} {\rm{Chem}}}^{1-24}$ and ${G}_{{\rm{GEOS}} \mbox{-} {\rm{Chem}}}^{{\rm{overpass}}}$ are NO_2_ concentrations simulated by GEOS-Chem of daily average and that at satellite overpass time, respectively. Here we use the daily NO_2_ concentrations at 12:00 to replace the overpass time concentrations.

However, the satellite products cannot obtain the atmospheric gaseous HNO_3_, and particulate NO_3_^−^ and NH_4_^+^. Based on ground measurements or the simulation results on NO2, HNO_3_, NO_3_^−^, NH_3_ and NH_4_^+^, the linear relationships were constructed between NO_2_, gaseous HNO_3_ and particulate NO_3_^−^, and between NH_3_ and NH_4_^+^, on monthly and annual scales^6,19,28^. Using these linear relationships and the remotely sensed estimation on surface NO_2_ concentrations, the monthly averages of the surface concentrations of NO_3_^−^ and HNO_3_ can be deduced. In this study, we used the surface IN concentration relationships simulated by GEOS-Chem and the satellite-estimated surface NH_3_ and NO_2_ concentrations to derive the surface gaseous HNO_3_, particulate NO_3_^−^ and NH_4_^+^ concentrations^24,28^.

To determine the V_d_ in the inferential model, a Big-leaf model was used to simulate the deposition rates of the major N fractions. It can be expressed as^29^:

6 $${{\rm{V}}}_{{\rm{d}}}=\frac{1}{{{\rm{r}}}_{{\rm{a}}}+{{\rm{r}}}_{{\rm{b}}}+{{\rm{r}}}_{{\rm{c}}}}+{{\rm{V}}}_{{\rm{g}}}$$

where r_a_ is the aerodynamic resistance, r_b_ is the quasi-laminar boundary sub-layer resistance, r_c_ is the canopy resistance and V_g_ is the gravitational settling velocity.

For gases, the gravitational settling velocity can be ignored. The measurement of canopy resistance is the most complex, and the basic starting point is to generalize the crop canopy as a “big leaf”, which is expressed as the combined stomatal resistance and soil resistance of each leaf according to the “big leaf theory”^30^. In this study, an inferential model was used to quantify dry deposition as two components: surface nitrogen fraction concentration and deposition rate.

### Wet deposition model

The conventional “bottom-up” method to estimate IN wet deposition by CTM contains clear in-cloud, sub-cloud and precipitation processes, which relies on the emission inventory and accuracy of pattern reflection and transmission. In this study, a top-down estimation method based on satellite data was used by constructing a wet deposition model (mixed-effects model) with mixed-layer NO_2_ and NH_3_ concentrations, nitrate and ammonia wet deposition from sites observations and precipitation data. This method has two key steps below:

1. Convert the N concentrations from troposphere to atmospheric boundary layer
  The clear process of IN wet deposition usually starts at the rainfall height rather than the top troposphere height. It is more reasonable that the NO_2_ and NH_3_ concentrations below the rainfall height should be used to build the wet deposition model^6,31^. As rainfall heights could not be performed and nitrogen components were mainly within the atmospheric boundary layer (ABL), the N concentrations in the atmospheric boundary layer heights were used here instead^32^. The main conversion is expressed as:
  7 $${S}_{ABL}={S}_{{\rm{trop}}}\times \frac{\varphi ({h}_{ABL})}{\varphi ({h}_{{\rm{trop}}})}$$
  8 $$\varphi ({h}_{{\rm{ABL}}})={\int }_{0}^{{h}_{{\rm{ABL}}}}\rho (Z){\rm{d}}x$$
  Where *S*_*ABL*_ is the ABL NO_2_ or NH_3_ columns, and *S*_trop_ is the tropospheric NO_2_ or NH_3_ columns from satellite. $\frac{\varphi \left({h}_{ABL}\right)}{\varphi \left({h}_{{\rm{trop}}}\right)}$ is the ratio of the model-simulated ABL NO_2_ or NH_3_ column to its tropospheric column concentration, where $\varphi \left({h}_{{\rm{ABL}}}\right)$ can be obtained from the integration of the profile function from 0 to the top of the atmospheric boundary layer (*h*_ABL_).
2. Develop a linear mixed-effects model monthly scale,using ABL NO_2_ and NH_3_ columns from satellite, precipitation data, and sites observations on IN wet deposition (NO_3_^–^-N and NH_4_^+^-N)^33^.The model combines fixed effects, which generally refer to the response variable (dependent variable); and random effects, which refer to the relevant influencing factor variables. In this study, the model to estimate wet deposition of nitrate or ammonia N was followed^34^:

9 $${N}_{ij}={\alpha }_{{\rm{j}}}+{\beta }_{{\rm{i}}}\times {P}_{{\rm{ij}}}\times {({S}_{ABL})}_{{\rm{ij}}}+{\varepsilon }_{{\rm{ij}}}$$

Where *N*_*ij*_ is wet NO_3_^−^ or NH_4_^+^ deposition at month i and site j; *P*_ij_ is precipitation and (S_ABL_)_ij_ means the mixed layer NO_2_ or NH_3_ column concentration at station j in month i. *P*_ij_×(S_ABL_)_ij_ represents an indicator of the combined effect of atmospheric precipitation and inorganic nitrate-N or ammonia-N compounds. *β*_i_ and *α*_j_ are the slope and intercept of random effects, representing seasonal variability and spatial effects. *ε*_ij_ represents the random error at month i and site j.

## Data Records

The atmospheric inorganic nitrogen deposition fluxes in China database from 2001 to 2020 is available at 10.6084/m9.figshare.24057120.v3^20^. The database consists three files: the ‘data file’ is the main file, includes deposition fluxes of dry, wet and total IN. The ‘readme file’ and the ‘source file’ explains the description, units, and the full references used in the database.

## Technical Validation

**Fig. 2:**
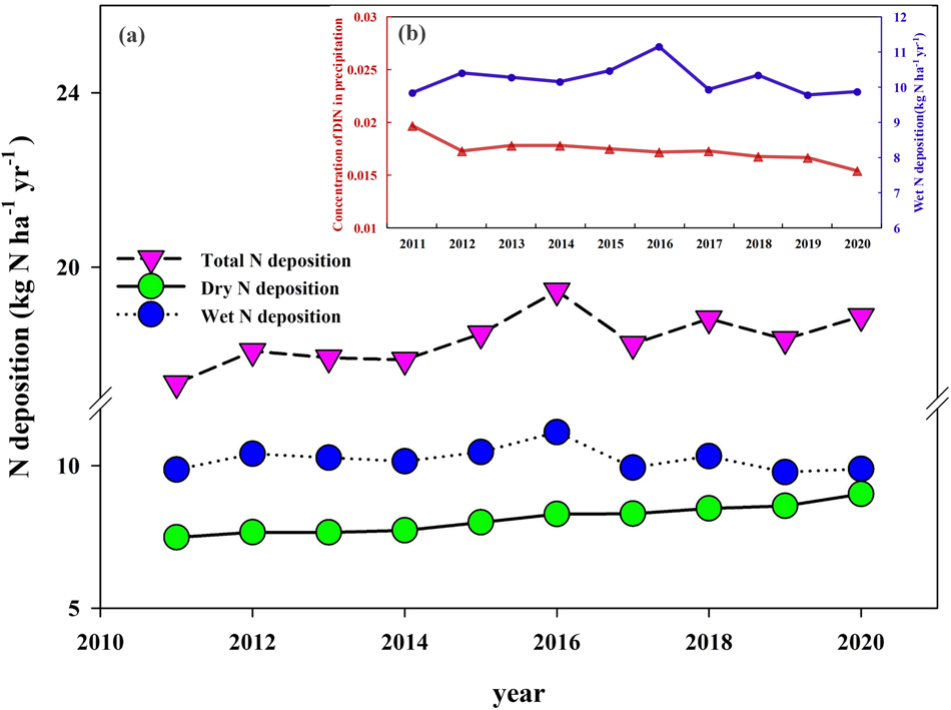
Temporal variations of the dry, wet and total N deposition across China during 2011–2020. (b) Ten-year variations of the estimated wet N deposition flux and concentration of DIN in precipitation obtained by removing the rainfall effect during 2011−2020.

In terms of the temporal trend, there is a small increase in the total IN deposition over land in China during 2011–2020 (Fig. 2a). The dry deposition fluxes had a weak upward trend over the ten-year period, but the fluctuation was small. The wet deposition fluxes fluctuated up and down between years due to the influence of rainfall^35,36^, but showed a clear decreasing trend in the latter five years. This trend, transitioning from growth to stabilization to decline, reflected the role and process of policy control and emission reduction measures. In order to more accurately investigate the temporal trends in N deposition fluxes, we should remove the influence of the rainfall amount^37^. In this work, the ratio of the wet N deposition fluxes and precipitation, namely concentration of dissolved inorganic nitrogen (DIN) in precipitation, was selected as an indicator to reflect the temporal variability of the N deposition fluxes after removing the effect of the rainfall amount (Fig. 2b). The wet N deposition fluxes displayed decreased temporal trend during 2011−2020, which was consistent with the trend presented by the NNDMN sites^9^. In the previous study, ammonia-N showed an increasing trend and nitrate-N showed a decreasing trend from 2008 to 2017, and the ratio of reduced to oxidized N deposition in China increased since 2011^22^. Even though nitrate-N decreased significantly from year to year due to the strict control of nitrogen oxides in the 12th Five-Year Plan^38^, the trend of total N still showed a certain increase, which was also confirmed in this study.

Quantitatively, the annual averages of total IN deposition in China ranged from 16 to 22 kg N ha^−1^. The ranges of annual averages of dry deposition was 7–10 kg N ha^−1^ and the wet deposition was 9–12 kg N ha^−1^. The proportion of wet deposition accounted for a higher percentage of total deposition than dry deposition in China. However, the contribution from dry deposition gradually increased over time. These results demonstrated the critical role of dry deposition in estimating total IN deposition^8^.

**Fig. 3:**
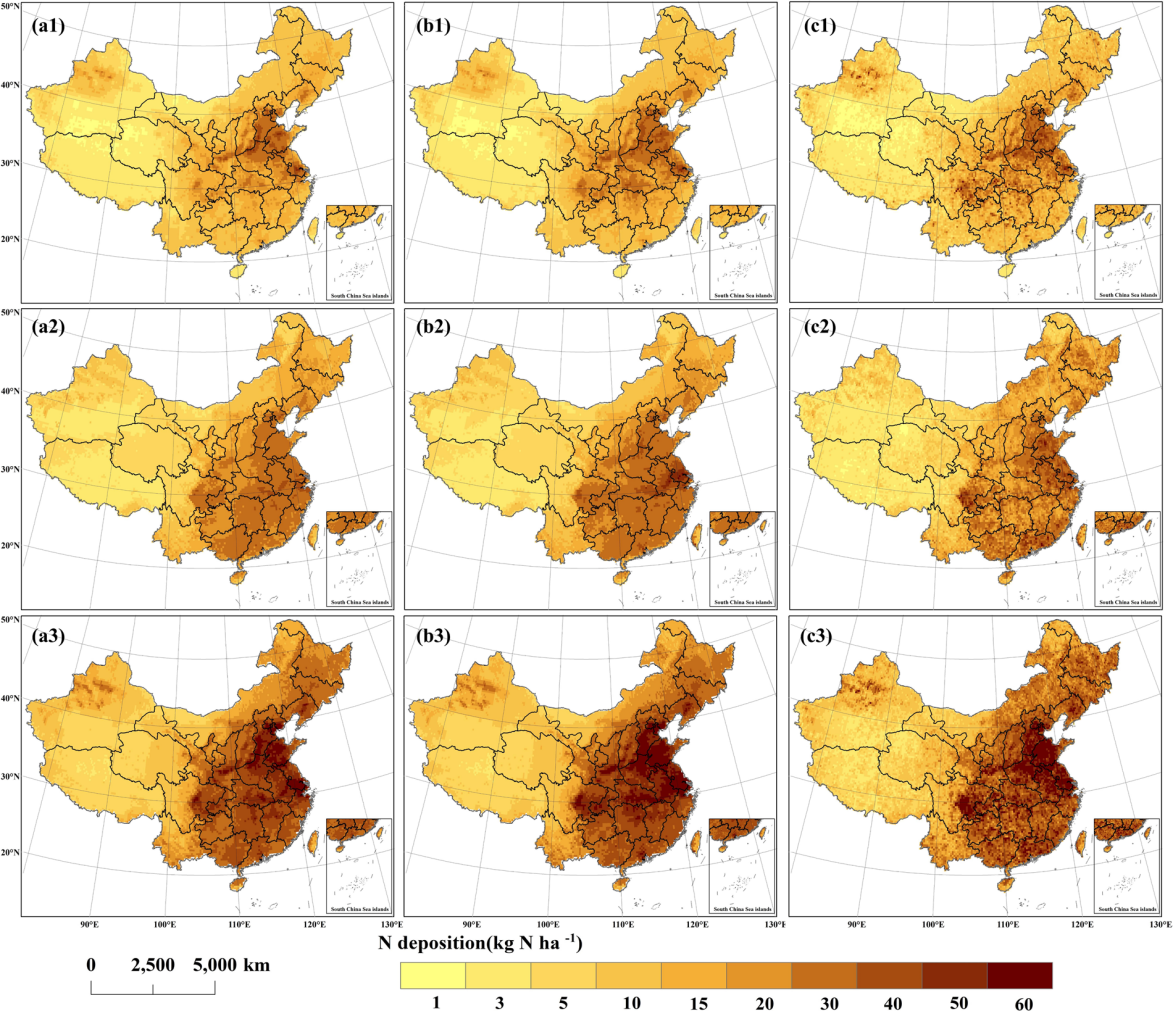
Spatial patterns of atmospheric IN deposition over China in 2012, 2016 and 2018. (a1-a3) spatial distribution of dry, wet, and total IN deposition in 2012, respectively. (b1-b3) spatial distribution of dry, wet, and total IN deposition in 2016, respectively. (c1-c3) spatial distribution of dry, wet, and total IN deposition in 2018, respectively. The deposition fluxes are presented hierarchically.

The spatial distributions of dry, wet, and total N deposition fluxes are described in Fig. 3. For dry deposition, Eastern China (including Shandong, Hebei, Henan, Jiangsu, Beijing, Tianjin, Xi’an, Taiyuan, Chengdu, Chongqing, etc.) had the highest values of dry deposition, followed by the regions in eastern and southeast China, central Liaoning, and northwest China. Low values were mainly distributed in western China (Tibet, Qinghai), northwest China (southeast Xinjiang, northwestern Inner Mongolia), etc. The high values of wet deposition were mainly concentrated in Jiangsu, Anhui, and eastern Hubei provinces, as well as Chengdu and Chongqing in Sichuan Province, Hebei, Henan, and western Guangdong Province, and Beijing. The low values were mainly concentrated in western China (Xinjiang, Tibet, Qinghai, northwestern Inner Mongolia, etc.) and were in high agreement with the spatial variations of precipitation. There were large differences in dry and wet deposition ratios in different regions, with Sichuan, Chongqing, and Guangdong mainly attributed to wet deposition because of higher precipitation in southern regions compared to northern regions of China. Overall, the spatial heterogeneity of atmospheric IN deposition is large, mainly characterized by lower values in the western region and higher values in the eastern region.

In our previous studies^26^, the accuracy of the simulated profiles of NH_3_ and NO_2_ concentrations by Gaussian functions had been evaluated, with the R^2^ above 0.95 for more than 99% of the grid points. Based on the concentrations collected from observation sites, the point-to-point validation on the remotely sensed estimates of near-surface NH_3_ and NO_2_ concentrations was performed with R^2^ of 0.72 and 0.71, respectively. Furthermore, the regression correlation coefficients for the wet deposition NH_4_^+^ and NO_3_^-^ mixed effects models reached to 0.81 and 0.87, respectively^6,22,24,27^.

In addition, the estimates of atmospheric IN deposition fluxes were within a reasonable range compared to previous studies^8,10^. The high IN deposition region of the North China Plain shown in this study has been consistently found in studies by other scholars^7,35,39^. The percentage of dry deposition gradually increased during 2011–2020, which was consistent with the results from the previous studies^8,40^.

## Data Availability

The inorganic nitrogen deposition database1.0 describes the data is available at Figshare data record (10.6084/m9.figshare.24057120.v3)^20^.
